# New strategies for profiling and characterization of human milk oligosaccharides

**DOI:** 10.1093/glycob/cwaa028

**Published:** 2020-04-02

**Authors:** Sara Porfirio, Stephanie Archer-Hartmann, G Brett Moreau, Girija Ramakrishnan, Rashidul Haque, Beth D Kirkpatrick, William A Petri, Parastoo Azadi

**Affiliations:** 2 Complex Carbohydrate Research Center, The University of Georgia, Athens, GA 30602, USA; 3 Department of Medicine/Infectious Diseases, University of Virginia, Charlottesville, VA 22903, USA; 4 International Centre for Diarrhoeal Disease Research, Bangladesh (icddr,b), Dhaka 1212, Bangladesh; 5 Department of Medicine, University of Vermont, Burlington, VT 05401, USA

**Keywords:** CID, human milk oligosaccharides (HMOs), LC-NSI-MS/MS, MALDI-TOF-MS, structural analysis

## Abstract

Human breast milk is an incredibly rich and complex biofluid composed of proteins, lipids and complex carbohydrates, including a diverse repertoire of free human milk oligosaccharides (HMOs). Strikingly, HMOs are not digested by the infant but function as prebiotics for bacterial strains associated with numerous benefits. Considering the broad variety of beneficial effects of HMOs, and the vast number of factors that affect breast milk composition, the analysis of HMO diversity and complexity is of utmost relevance. Using human milk samples from a cohort of Bangladeshi mothers participating in a study on malnutrition and stunting in children, we have characterized breast milk oligosaccharide composition by means of permethylation followed by liquid chromatography coupled with high-resolution tandem mass spectrometry (LC-MS/MS) analysis. This approach identified over 100 different glycoforms and showed a wide diversity of milk composition, with a predominance of fucosylated and sialylated HMOs over nonmodified HMOs. We observed that these samples contain on average 80 HMOs, with the highest permethylated masses detected being >5000 mass units. Here we report an easily implemented method developed for the separation, characterization and relative quantitation of large arrays of HMOs, including higher molecular weight sialylated HMOs. Our ultimate goal is to create a simple, high-throughput method, which can be used for full characterization of sialylated and/or fucosylated HMOs. These results demonstrate how current analytical techniques can be applied to characterize human milk composition, providing new tools to help the scientific community shed new light on the impact of HMOs during infant development.

## Introduction

Breast milk is a vital biological fluid, providing 100% of mammalian infant nutrition at the beginning of life. While lipids, proteins and lactose act as major caloric nutrients (Tao et al. 2011; Oursel et al. 2017), breast milk of many species also contains considerable amounts (1–2%, w/v) of indigestible milk oligosaccharides (Smilowitz et al. 2014) that reach the neonate’s large intestine. Human milk oligosaccharides (HMOs) are a diverse group of free soluble oligosaccharides displaying a wide array of biological functions, acting as prebiotics (Newburg et al. 2005; Bode 2012), antiadhesive antimicrobials (Bode and Jantscher-Krenn 2012; Etzold and Bode 2014), immunomodulators (Zopf and Roth 1996; Kunz and Rudloff 2008; Bode 2015) and nutrient providers for the brain (Wang 2012). Concentrations of HMOs fluctuate with lactation stage (Kunz et al. 2000; Thurl et al. 2017), going from >20 g/L in colostrum to ~ 13 g/L in mature milk (Coppa et al. 1993; Gabrielli et al. 2011), and milk from mothers delivering prematurely has higher HMO concentration than term milk (Gabrielli et al. 2011). Even factors such as geographical location have been shown to affect HMO concentrations and profiles (McGuire et al. 2017). For these reasons, the study of breast milk, and particularly of HMOs, is currently one of the most active areas of research (Smilowitz et al. 2014). Despite the very well-known diversity and complexity of HMOs (Chen 2015), manufactured infant formulas are still very poor in HMO complexity, mostly because their chemical synthesis is often a cumbersome and laborious process that only recently generated asymmetric, multiantennary HMOs (Prudden et al. 2017). Thus, understanding HMO structural complexity and interpersonal variability is highly relevant not only for the development of better food supplements but also to increase our knowledge about the biochemistry of these oligosaccharides.

**Fig. 1:**
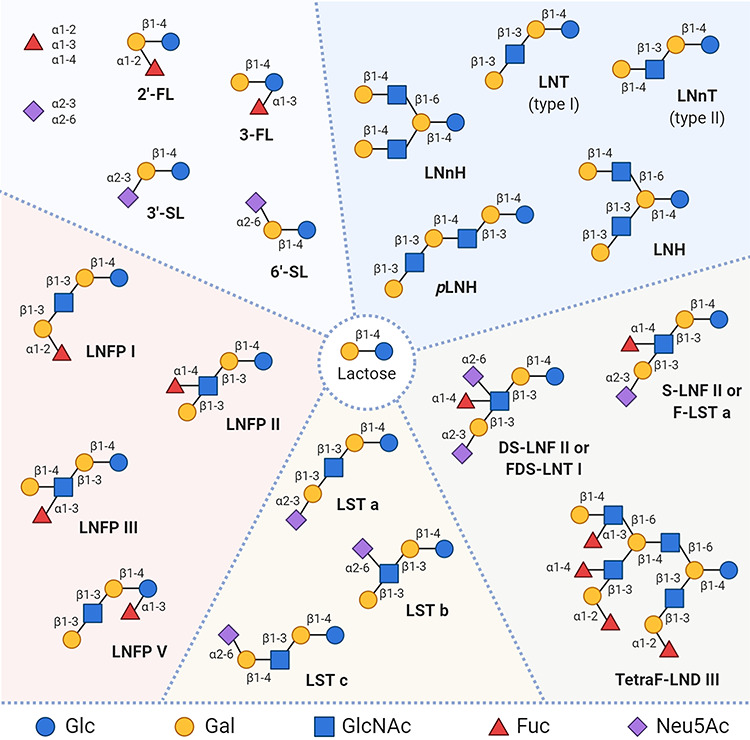
Structural diversity of human milk oligosaccharides. With very few exceptions, all HMOs are formed by a lactose core (Gal-β1 → 4-Glc, center), which can be extended enzymatically in repeats of lacto-*N*-biose (Gal-β1 → 3-GlcNAc) or *N*-acetyllactosamine (Gal-β1 → 4-GlcNAc) (upper right). Lactose can also be fucosylated or sialylated in different linkages (upper left). Linkage and position of fucose residues generate isomers, which can result in structures that are phenotypically related to secretor status and Lewis blood group (lower left). Isomerization can also occur as a result of sialylation of elongated core structures (lower center). HMOs can also be simultaneously fucosylated and sialylated, resulting in highly complex structures (lower right). The monosaccharide key is shown at the bottom. Abbreviations: 2′-FL, 2′-fucosyllactose; 3-FL, 3-fucosyllactose; 3′-SL, 3′-sialyllactose; 6′-SL, 6′-sialyllactose; LNFP I, II, III, V, lacto-*N*-fucopentaose I, II, III, V; LNT, lacto-*N*-tetraose; LNnT, lacto-*N*-neotetraose; LNH, lacto-*N*-hexaose; LNnH, lacto-*N*-neohexaose; *p*LNH, *para*-lacto-*N*-hexaose; LST a, b, c, sialyl-lacto-*N*-tetraoses a-c; S-LNF II or F-LST a, sialylfucosyllacto-*N*-tetraose; DS-LNF II or FDS-LNT I, fucosyldisialyllacto-*N*-tetraose; TetraF-LND III, tetrafucosyl-lacto-*N*-decaose III. Monosaccharide symbols follow the Symbol Nomenclature for Graphical Representation of Glycans (https://www.ncbi.nlm.nih.gov/glycans/snfg.html). This figure is available in black and white in print and in colour at *Glycobiology* online.

HMOs are complex glycans formed by glucose (Glc), galactose (Gal), *N*-acetylglucosamine (GlcNAc), fucose (Fuc) and *N*-acetylneuraminic acid (Neu5Ac). With very few exceptions (Kunz et al. 2000), all HMOs are formed by a reducing lactose core that can be extended enzymatically by lacto-*N*-biose (Galβ-1,3-GlcNAc, type 1 LacNAc) or *N*-acetyllactosamine (Gal-β1,4-GlcNAc, type 2 LacNAc) motifs. These structures can be further decorated by the addition of Fuc residues in α1,2-, α1,3-, and α1,4-linkages and/or Neu5Ac residues in α2,3- and α2,6-linkages, providing an array of Lewis structures and blood group antigens (Figure 1). The relative abundance of each group of HMOs has been described (Totten et al. 2012) and is related to the secretor status of the mother (Blank et al. 2012; Bode 2012), a phenotype associated with the type of fucosyltransferases expressed by an individual. Fucosylated and sialylated HMOs have been associated with microbial pathogen protection and child development outcomes, respectively (Chaturvedi et al. 2001; Charbonneau et al. 2016). Not only can HMOs be structurally complex but multiple positional and linkage isomers can also be found for the same monosaccharide composition (and thus mass), which further complicates their analysis. So far, over 200 individual HMO molecular species and more than 100 structures have been reported (Chen 2015; Urashima et al. 2018). Identification and structural assignment have been accomplished by a number of analytical techniques (reviewed in Ruhaak and Lebrilla 2012; Mantovani et al. 2016; Yan et al. 2017). Given its capability to resolve isomers, high pH anion-exchange chromatography with pulsed amperometric detection (HPAEC-PAD) has been used often to separate HMOs (Ruhaak and Lebrilla 2012 and references therein). However, prior separation of neutral and acidic glycans is needed. Reversed-phase high-performance liquid chromatography (RP-HPLC) is another widely used method for HMO analysis (Chaturvedi et al. 1997, 2001; Sumiyoshi et al. 2003; Morrow et al. 2004; Asakuma et al. 2008; Leo et al. 2009, 2010), although it requires sample derivatization since native HMOs are typically polar and are not retained by the column. HMOs have also been separated by hydrophilic interaction chromatography HPLC (HILIC) (Marino et al. 2011; Xu et al. 2017; Yan et al. 2018), which provides good isomer separation but requires oligosaccharide labeling, typically with 2-aminobenzamide using reductive amination. Other techniques such as micellar electrokinetic chromatography (MEKC) can provide good separation for native charged HMOs, but existing methods were developed for acidic HMOs and suffer from low sensitivity (Shen et al. 2000; Bao et al. 2007). Many different variants of capillary electrophoresis (CE) have also been applied to HMO analysis (Bao and Newburg 2008), although these typically require end-labeling to overcome the same limitation of sensitivity seen with MEKC. The main shortcoming of separation-based methods, aside from derivatization, is the need for standards to which retention times can be compared. In the case of HMOs, and depending on the specific structure, standards can be very expensive or nonexistent, thus making mass identification an almost obligatory tool for analysis. Mass-based methods can be used independently, frequently using MALDI-FTICR-MS (LoCascio et al. 2007; Ninonuevo et al. 2007; LoCascio et al. 2009) or coupled to HPLC separations. While offline approaches may be faster because no separation is needed, this in itself can be a major drawback if the samples are not completely pure and, moreover, isomer separation is not possible without prior column separation. Consequently, the most effective methods rely on LC separation of HMOs molecular species and MS ionization and fragmentation to characterize chemical structures. A noteworthy approach is nano-liquid chromatography chip-time-of-flight mass spectrometry (nano-LC chip-TOF MS) in positive mode, developed by the Lebrilla Lab. This method, which relies on a library containing retention time, mass and fragmentation information (Wu et al. 2010; Wu et al. 2011), allows for separation of >200 structures in a porous graphitized carbon (PGC) column and has been widely used in primate (Tao et al. 2011) and human samples (Totten et al. 2012; De Leoz et al. 2013; Wu et al. 2017), providing high-throughput and reproducible analysis (Totten et al. 2014). However, this method is fully dependent on in-house software and instrument stability which prevents its widespread use. Furthermore, the MS/MS analysis of native samples lacks structural information which is retained by permethylation. Permethylation is a widely used derivatization method, particularly in carbohydrate chemistry, which consists in the replacement of all hydrogens attached to oxygen and nitrogen atoms with methyl groups, providing higher sensitivity, better ionization and protection of labile groups (e.g. Neu5Ac), among other advantages (Costello et al. 2007). Currently, aside from electronic excitation dissociation (EED) and fixed-charge derivatization (Tang et al. 2018), only permethylation can enable MS^n^ to provide some sequence, branching and linkage information for glycans (Ashline et al. 2017). Emerging work from a NIST group (Remoroza et al. 2018), who developed a searchable, reference MS library of annotated oligosaccharides, was proposed as a method to identify unknown reduced (or nonreduced) HMOs, as well as, potentially, permethylated HMOs based on spectral matching. Impressively, this library currently contains 469 positive and negative ion spectra resulting from by HILIC-ESI-MS/MS analysis and will be an exciting option for future work. However, the database is still built upon existing standards, and high molecular weight (11–12 sugar units) oligosaccharides had been only partially identified at the time of publication.

While some reports have previously described LC-MS separation of permethylated HMOs (Dong et al. 2016; Oursel et al. 2017), these methods focus on the most abundant, low molecular weight, species and only describe a fairly limited number of HMOs. Here we present a much improved nanoliquid chromatography nanospray tandem mass spectrometry (nLC-NSI-MS/MS) method for HMOs analysis, using reversed-phase chromatography of permethylated samples and collision-induced dissociation (CID) MS/MS fragmentation. This approach also includes oligosaccharide profiling by matrix-assisted laser-induced time-of-flight mass spectrometry (MALDI-TOF-MS). We show how this method can be applied to milk glycomic profiling and how fragmentation of permethylated HMOs can provide structural information and isomer distinction.

## Results and Discussion

### HMO profiling by MALDI-TOF-MS analysis

**Fig. 2:**
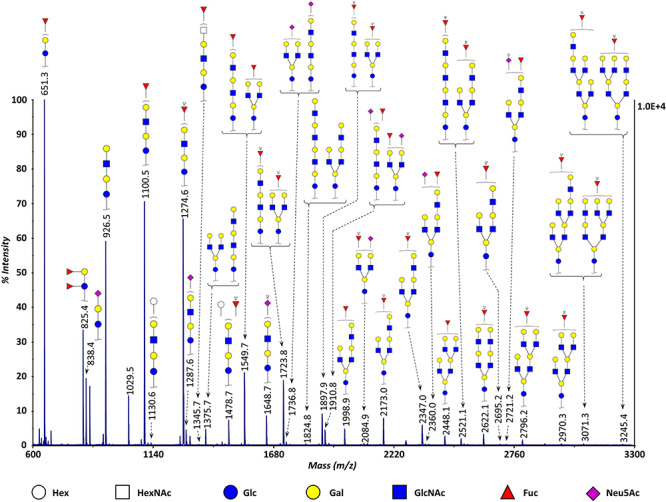
Representative MALDI-TOF-MS spectrum of permethylated human milk oligosaccharides. All masses correspond to fully permethylated, free reducing end, sodium adducts of HMOs. Possible structures for each mass are shown. Free lactose was excluded from the spectrum. HMOs were identified using Glycomod (web.expasy.org/glycomod) as a search engine. This figure is available in black and white in print and in colour at *Glycobiology* online.

Permethylated samples were profiled initially by MALDI-TOF-MS to characterize major HMOs present in human milk in terms of mass and monosaccharide composition (Figure 2). MALDI-TOF-MS analysis revealed the expected complexity of breast milk. Using this approach, up to 32 of the highest abundance permethylated masses were identified in human milk. While the number of HMO structures described in the literature is much higher than this (Chen 2015), since structural isomers do not differ in terms of mass, the total number of glycoforms present in this spectrum is in reality much higher. This is displayed in Figure 2 by the different possible structures for the same mass (shown in brackets). In addition, many of the high molecular weight species are present in human milk at very low abundances (<1%, Tao et al. 2011), which makes their detection by MALDI-TOF-MS more difficult. In agreement with previous reports (Totten et al. 2014; Chen 2015; Charbonneau et al. 2016), the samples contained a wide variety of fucosylated and/or sialylated HMOs, with increasing degrees of complexity. It should be noted here that while free lactose is the main oligosaccharide of these samples (see ahead), it is not considered a true HMO because, unlike HMOs, it is readily digested by the infant gut, thus being a primary source of nutrition (Newburg 2013). Different approaches have been used to remove free lactose from human milk, from size-exclusion chromatography using Sephadex G-25 (Marino et al. 2011) and small scale PGC cartridges (Xu et al. 2017), to large scale simulated moving bed chromatography (Mank et al. 2019). While lactose removal can help generate cleaner and more purified HMO samples, it also increases the chances of HMO loss and bias, which is especially relevant for most of the low abundance species. This is detrimental to breast milk studies because although the 10 most abundant HMOs make up ~ 75% of the total HMO mass (Grabarics et al. 2017), the greatest structural (and likely functional) diversity of HMOs resides in the 25% of high molecular weight glycoforms. For this reason, we chose not to remove lactose and take advantage of a selected mass window in the case of MALDI-TOF-MS and chromatographic separation in the case of LC-MS (see ahead).

### nLC-NSI-MS/MS analysis of permethylated milk oligosaccharides

**Fig. 3:**
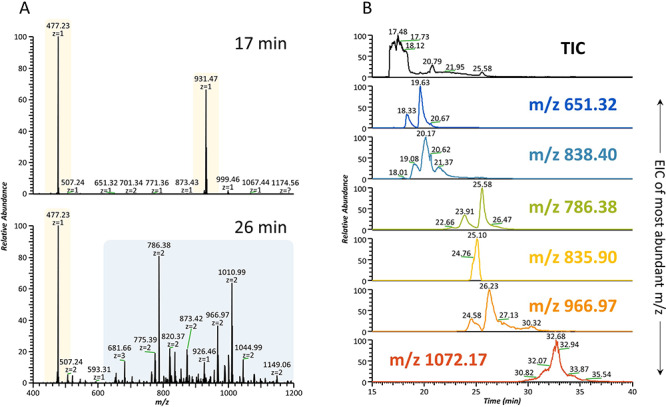
LC-MS separation of permethylated human milk oligosaccharides. (**A**) Full MS scans at 17 min and 26 min of elution time. For the first 5 min of the separation, the main eluting compound is free lactose (top). After 20 min (bottom), HMOs start to elute and can be isolated from lactose. Several multiply charged ions can be seen. (**B**) EICs corresponding to the most abundant charge state of some of the most abundant HMOs found in the samples. The TIC trace (shown on top) shows the full elution profile, where the main peak corresponds to the elution of lactose (full MS shown in **A**). Mass accuracy: 5 ppm. This figure is available in black and white in print and in colour at *Glycobiology* online.

Considering the limitations of MALDI-TOF-MS, and to allow for HMO isolation from lactose, the samples were further separated and analyzed by nLC-NSI-MS/MS (Figure 3). Given the overwhelming abundance of free lactose in relation to HMOs, the first 5 min of the separation result almost exclusively in the ionization of lactose (*m*/*z* 477.23) and lactose dimers that are formed during ionization (*m*/*z* 931.47) as a consequence of high concentration (Figure 3A). After 20 min, and even though lactose is still the main ion present, HMOs start to elute (Figure 3A and B), up until 35 min. The benefits of LC separation are illustrated in Figure 3B, where we can see the extracted ion chromatograms (EICs) of some of the most abundant HMOs, and although they are not fully resolved, it is clear that reasonable separation can be achieved. When using electro/nanospray ionization, several multiply charged ions can be observed for the same mass, making data analysis much more complex. An example of this is the high number of doubly and triply charged ions visible in Figure 3A. However, LC-NSI-MS methods are also associated with higher sensitivity, allowing for the detection of a considerably higher number of glycoforms than MALDI-TOF-MS. In fact, using this method, a total of 102 HMOs permethylated masses were detected (Table I), including new HMOs that have not been described in the literature. Specifically, we were able to detect HMOs of higher molecular weights than previously reported using commercially available instrumentation and columns that are common to most academic laboratories, a considerable improvement over previous methods (Dong et al. 2016). It should be noted here that this list corresponds to oligosaccharide *masses*, and as mentioned before, many HMOs have structural isomers, implying that the number of *structures* found in these samples is much higher than this. Table I shows the full list of HMOs detected in the human samples analyzed in this study. The list of HMOs detected in each sample can be found in the Supplementary Table SI. Full separation of HMO peaks is not possible under these conditions, which is to be expected given sample complexity. It is true that the separation of native HMOs on PGC columns is better than that of permethylated HMOs in C18 columns (Oursel et al. 2017), and while PGC columns have even been used for the separation of permethylated *N*-glycans (Costello et al. 2007; Zhou et al. 2017), they are better suited to native oligosaccharide separations, and are not as widely available as reversed phase columns. However, while separation is definitely important, especially in terms of isomer distinction, permethylation is crucial in increasing sensitivity by facilitating ionization, as well as in providing relevant structural information.

**Table I:** Full list of HMOs (not including isomers) detected in human milk samples by nLC-NSI-MS/MS analysis

Permethylated theoretical mass [M + Na]^+^	Native reduced mass [M]	Most abundant charge state	Monosaccharide composition
651.3200	490.1897	1	Hex_2_ Fuc_1_
722.3572	547.2112	1	Hex_2_ HexNAc_1_
825.4092	636.2476	1	Hex_2_ Fuc_2_
838.4045	635.2272	1	Hex_2_ NeuAc_1_
**896.4464**	**693.2691**	**1**	**Hex**_**2**_ **HexNAc**_**1**_ **Fuc**_**1**_
926.4570	709.2640	1	Hex_3_ HexNAc_1_
1100.5462	855.3219	1	Hex_3_ HexNAc_1_ Fuc_1_
1130.5568	871.3168	1	Hex_4_ HexNAc_1_
1274.6354	1001.3798	1	Hex_3_ HexNAc_1_ Fuc_2_
1287.6307	1000.3594	1	Hex_3_ HexNAc_1_ NeuAc_1_
**1304.6460**	**1017.3747**	**1**	**Hex**_**4**_ **HexNAc**_**1**_ **Fuc**_**1**_
1345.6726	1058.4013	1	Hex_3_ HexNAc_2_ Fuc_1_
1375.6832	1074.3962	1	Hex_4_ HexNAc_2_
1461.7199	1146.4173	1	Hex_3_ HexNAc_1_ Fuc_1_ NeuAc_1_
**1478.7352**	**1163.4326**	**1**	**Hex**_**4**_ **HexNAc**_**1**_ **Fuc**_**2**_
**1491.7305**	**1162.4122**	**1**	**Hex**_**4**_ **HexNAc**_**1**_ **NeuAc**_**1**_
1519.7618	1204.4592	1	Hex_3_ HexNAc_2_ Fuc_2_
1549.7724	1220.4541	2	Hex_4_ HexNAc_2_ Fuc_1_
**1579.7830**	**1236.4490**	**2**	**Hex**_**5**_ **HexNAc**_**2**_
1648.8045	1291.4548	2	Hex_3_ HexNAc_1_ NeuAc_2_
**1706.8464**	**1349.4967**	**2**	**Hex**_**3**_ **HexNAc**_**2**_ **Fuc**_**1**_ **NeuAc**_**1**_
1723.8617	1366.5120	2	Hex_4_ HexNAc_2_ Fuc_2_
1736.8569	1365.4916	2	Hex_4_ HexNAc_2_ NeuAc_1_
1753.8722	1382.5069	2	Hex_5_ HexNAc_2_ Fuc_1_
1822.8937	1437.5127	2	Hex_3_ HexNAc_1_ Fuc_1_ NeuAc_2_
1824.9094	1439.5284	2	Hex_5_ HexNAc_3_
1897.9509	1512.5699	2	Hex_4_ HexNAc_2_ Fuc_3_
1910.9462	1511.5495	2	Hex_4_ HexNAc_2_ Fuc_1_ NeuAc_1_
1927.9615	1528.5648	2	Hex_5_ HexNAc_2_ Fuc_2_
**1940.9567**	**1527.5444**	**2**	**Hex**_**5**_ **HexNAc**_**2**_ **NeuAc**_**1**_
1998.9986	1585.5863	2	Hex_5_ HexNAc_3_ Fuc_1_
2085.0354	1657.6074	2	Hex_4_ HexNAc_2_ Fuc_2_ NeuAc_1_
2098.0307	1656.5870	2	Hex_4_ HexNAc_2_ NeuAc_2_
**2102.0507**	**1674.6227**	**2**	**Hex**_**5**_ **HexNAc**_**2**_ **Fuc**_**3**_
**2115.0460**	**1673.6023**	**2**	**Hex**_**5**_ **HexNAc**_**2**_ **Fuc**_**1**_ **NeuAc**_**1**_
2173.0879	1731.6442	2	Hex_5_ HexNAc_3_ Fuc_2_
2203.0984	1747.6391	2	Hex_6_ HexNAc_3_ Fuc_1_
2272.1199	1802.6449	2	Hex_4_ HexNAc_2_ Fuc_1_ NeuAc_2_
2347.1771	1877.7021	2	Hex_5_ HexNAc_3_ Fuc_3_
2360.1724	1876.6817	2	Hex_5_ HexNAc_3_ Fuc_1_ NeuAc_1_
**2377.1877**	**1893.6970**	**2**	**Hex**_**6**_ **HexNAc**_**3**_ **Fuc**_**2**_
2448.2249	1950.7185	2	Hex_6_ HexNAc_4_ Fuc_1_
2459.2044	1947.6824	2	Hex_4_ HexNAc_2_ NeuAc_3_
2521.2664	2023.7600	2	Hex_5_ HexNAc_3_ Fuc_4_
2534.2616	2022.7396	2	Hex_5_ HexNAc_3_ Fuc2 NeuAc_1_
2547.2569	2021.7192	2	Hex_5_ HexNAc_3_ NeuAc_2_
2622.3141	2096.7764	2	Hex_6_ HexNAc_4_ Fuc_2_
2635.3094	2095.7560	2	Hex_6_ HexNAc_4_ NeuAc_1_
2695.3556	2169.8179	2	Hex_5_ HexNAc_3_ Fuc_5_
2708.3509	2168.7975	2	Hex_5_ HexNAc_3_ Fuc_3_ NeuAc_1_
2721.3461	2167.7771	2	Hex_5_ HexNAc_3_ Fuc_1_ NeuAc_2_
2796.4033	2242.8343	2	Hex_6_ HexNAc_4_ Fuc_3_
2809.3986	2241.8139	2	Hex_6_ HexNAc_4_ Fuc_1_ NeuAc_1_
**2895.4354**	**2313.8350**	**2**	**Hex**_**5**_ **HexNAc**_**3**_ **Fuc**_**2**_ **NeuAc**_**2**_
2970.4926	2388.8922	2	Hex_6_ HexNAc_4_ Fuc_4_
2983.4878	2387.8718	2	Hex_6_ HexNAc_4_ Fuc_2_ NeuAc_1_
2996.4831	2386.8514	2	Hex_6_ HexNAc_4_ NeuAc_2_
**3069.5246**	**2459.8929**	**2**	**Hex**_**5**_ **HexNAc**_**3**_ **Fuc**_**3**_ **NeuAc**_**2**_
3071.5403	2461.9086	2	Hex_7_ HexNAc_5_ Fuc_2_
**3082.5199**	**2458.8725**	**3**	**Hex**_**5**_ **HexNAc**_**3**_ **Fuc**_**1**_ **NeuAc**_**3**_
**3099.5352**	**2475.8878**	**3**	**Hex**_**6**_ **HexNAc**_**3**_ **Fuc**_**2**_ **NeuAc**_**2**_
3144.5818	2534.9501	3	Hex_6_ HexNAc_4_ Fuc_5_
3157.5771	2533.9297	3	Hex_6_ HexNAc_4_ Fuc_3_ NeuAc_1_
3170.5724	2532.9093	3	Hex_6_ HexNAc_4_ Fuc_1_ NeuAc_2_
3245.6296	2607.9665	3	Hex_7_ HexNAc_5_ Fuc_3_
**3269.6044**	**2603.9100**	**3**	**Hex**_**5**_ **HexNAc**_**3**_ **NeuAc**_**4**_
**3273.6244**	**2621.9457**	**3**	**Hex**_**6**_ **HexNAc**_**3**_ **Fuc**_**3**_ **NeuAc**_**2**_
**3275.6401**	**2623.9614**	**3**	**Hex**_**8**_ **HexNAc**_**5**_ **Fuc**_**2**_
**3331.6663**	**2679.9876**	**3**	**Hex**_**6**_ **HexNAc**_**4**_ **Fuc**_**4**_ **NeuAc**_**1**_
**3344.6616**	**2678.9672**	**3**	**Hex**_**6**_ **HexNAc**_**4**_ **Fuc**_**2**_ **NeuAc**_**2**_
3419.7188	2754.0244	3	Hex_7_ HexNAc_5_ Fuc_4_
3432.7141	2753.0040	3	Hex_7_ HexNAc_5_ Fuc_2_ NeuAc_1_
**3443.6936**	**2749.9679**	**3**	**Hex**_**5**_ **HexNAc**_**3**_ **Fuc**_**1**_ **NeuAc**_**4**_
**3449.7294**	**2770.0193**	**3**	**Hex**_**8**_ **HexNAc**_**5**_ **Fuc**_**3**_
**3518.7508**	**2825.0251**	**3**	**Hex**_**6**_ **HexNAc**_**4**_ **Fuc**_**3**_ **NeuAc**_**2**_
**3531.7461**	**2824.0047**	**3**	**Hex**_**6**_ **HexNAc**_**4**_ **Fuc**_**1**_ **NeuAc**_**3**_
3593.8080	2900.0823	3	Hex_7_ HexNAc_5_ Fuc_5_
**3595.7873**	**2874.0302**	**3**	**Hex**_**9**_ **HexNAc**_**4**_ **Fuc**_**2**_ **NeuAc**_**1**_
3606.8033	2899.0619	3	Hex_7_ HexNAc_5_ Fuc_3_ NeuAc_1_
**3617.7829**	**2896.0258**	**3**	**Hex**_**5**_ **HexNAc**_**3**_ **Fuc**_**2**_ **NeuAc**_**4**_
**3619.7986**	**2898.0415**	**3**	**Hex**_**7**_ **HexNAc**_**5**_ **Fuc**_**1**_ **NeuAc**_**2**_
**3692.8401**	**2971.0830**	**3**	**Hex**_**6**_ **HexNAc**_**4**_ **Fuc**_**4**_ **NeuAc**_**2**_
3694.8558	2973.0987	3	Hex_8_ HexNAc_6_ Fuc_3_
**3705.8354**	**2970.0626**	**3**	**Hex**_**6**_ **HexNAc**_**4**_ **Fuc**_**2**_ **NeuAc**_**3**_
**3718.8306**	**2969.0422**	**3**	**Hex**_**6**_ **HexNAc**_**4**_ **NeuAc**_**4**_
**3793.8878**	**3044.0994**	**3**	**Hex**_**7**_ **HexNAc**_**5**_ **Fuc**_**2**_ **NeuAc**_**2**_
3868.9450	3119.1566	3	Hex_8_ HexNAc_6_ Fuc_4_
3881.9403	3118.1362	3	Hex_8_ HexNAc_6_ Fuc_2_ NeuAc_1_
**3892.9199**	**3115.1001**	**3**	**Hex**_**6**_ **HexNAc**_**4**_ **Fuc**_**1**_ **NeuAc**_**4**_
**3967.9771**	**3190.1573**	**3**	**Hex**_**7**_ **HexNAc**_**5**_ **Fuc**_**3**_ **NeuAc**_**2**_
**4043.0343**	**3265.2145**	**3**	**Hex**_**8**_ **HexNAc**_**6**_ **Fuc**_**5**_
4056.0295	3264.1941	3	Hex_8_ HexNAc_6_ Fuc_3_ NeuAc_1_
**4142.0663**	**3336.2152**	**3**	**Hex**_**7**_ **HexNAc**_**5**_ **Fuc**_**4**_ **NeuAc**_**2**_
**4217.1235**	**3411.2724**	**3**	**Hex**_**8**_ **HexNAc**_**6**_ **Fuc**_**6**_
4230.1188	3410.2520	3	Hex_8_ HexNAc_6_ Fuc_4_ NeuAc_1_
**4243.1140**	**3409.2316**	**3**	**Hex**_**8**_ **HexNAc**_**6**_ **Fuc**_**2**_ **NeuAc**_**2**_
**4417.2033**	**3555.2895**	**3**	**Hex**_**8**_ **HexNAc**_**6**_ **Fuc**_**3**_ **NeuAc**_**2**_
**4692.3403**	**3774.3638**	**4**	**Hex**_**9**_ **HexNAc**_**7**_ **Fuc**_**2**_ **NeuAc**_**2**_
**4767.3975**	**3849.4210**	**4**	**Hex**_**10**_ **HexNAc**_**8**_ **Fuc**_**4**_
**4868.4452**	**3922.4374**	**4**	**Hex**_**11**_ **HexNAc**_**9**_ **Fuc**_**2**_
**5042.5344**	**4068.4953**	**4**	**Hex**_**11**_ **HexNAc**_**9**_ **Fuc**_**3**_
**5317.6714**	**4287.5696**	**4**	**Hex**_**12**_ **HexNAc**_**10**_ **Fuc**_**2**_

Permethylated masses correspond to sodium adducts. Native reduced masses are given for easier comparison with other references. Bold rows indicate HMOs that have not been previously reported in the literature.

### Importance of permethylation for HMO structure assignment

**Fig. 4:**
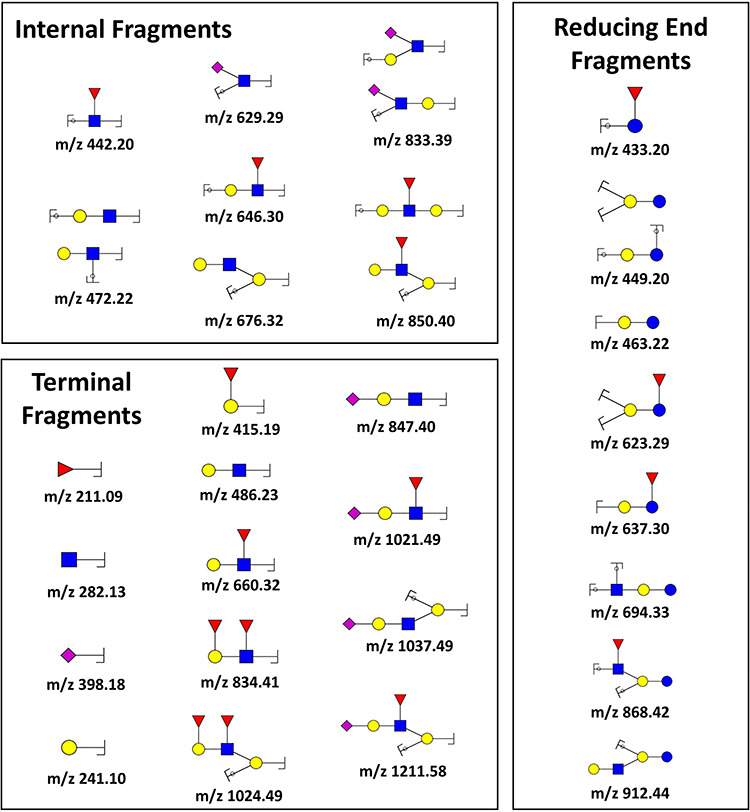
Examples of diagnostic fragment ions generated by permethylated HMOs. Permethylation provides structural information because it allows distinguishing internal, terminal and reducing end fragments. This figure is available in black and white in print and in colour at *Glycobiology* online.

Among many other advantages (Shajahan et al. 2017), permethylation provides diagnostic fragment ions (Figure 4), increasing the yield of useful structural information obtained from MS/MS experiments (Oursel et al. 2017). Permethylation aids in the distinction of internal fragments from terminal fragments (including reducing end vs. nonreducing end) and branched structures from linear structures, as well as in the determination of fucose position and linkage isomers. As interest in HMOs progresses from simple glycoforms, for which standards are available, to the more complex but biologically interesting forms, methodologies that do not automatically rely on standards will be needed. A particularly important advantage of permethylation is the fact that it precludes the occurrence of “internal residue loss” (Kovácik et al. 1995; Harvey et al. 2002), a phenomenon characterized by rearrangement reactions involving migration of fucose, and sometimes other monosaccharide residues (Kovácik et al. 1995), typically found in CID spectra of native glycans or even glycans derivatized at their reducing end (e.g. 2-AB, 3-AQ). This migration is known to happen in *N*- and *O*-glycans, as well as HMOs (Harvey et al. 2002; Zhou et al. 2017), and is affected by the type of adduct ions (Ernst et al. 1997; Brüll et al. 1998), aglycon (Ma et al. 2000), and derivatization (Brüll et al. 1997; Franz and Lebrilla 2002; Wuhrer et al. 2006; Nwosu et al. 2015). Experiments by Wuhrer et al. (2006) found that fragmentation of fucosylated *N*-glycans resulted in additional fragmentation ions that had acquired fucose residues from other parts of the oligosaccharide. This transfer was found on native reducing or 2-AB labeled *N*-glycans with protonated charge carriers. It was also observed by both ion-trap-based CID as well as laser-induced dissociation via MALDI-TOF/TOF, even though both fragmentation methods occur under drastically different ionic lifetimes, suggesting that fucose transfer is an active part of the decomposition. This included the observed transfer of an effective fucose onto an existing Lewis X antenna which would improperly suggest a Lewis Y epitope. A similar outcome had previously been reported by Ernst et al. (1997), who described the formation of “false” sugar sequence ions from branched sialyl-Lewis-type tetrasaccharides as a result of fucose migration towards sialic acid residues in both [M + H]^+^ and [M + NH_4_]^+^ adduct ions.

More importantly, while these rearrangements have long been associated with activation during CID fragmentation in tandem MS experiments, recent work by Mucha et al. (2018) using cold-ion spectroscopy has shown that fucose migration is not limited to fragments obtained in the gas phase during tandem MS, but it can also occur in intact glycan ions. The exact mechanism by which this process occurs is still not fully understood, although it is known that the reaction happens in the presence of a proton and is inhibited in its absence (thus, it is typically not observed with sodium adducts (Brüll et al. 1998; Kovacik et al. 1998; Franz and Lebrilla 2002)), and recent data have demonstrated that the mobility of the proton, not CID fragmentation, is a necessary prerequisite for migration reactions (Lettow et al. 2019).

**Fig. 5:**
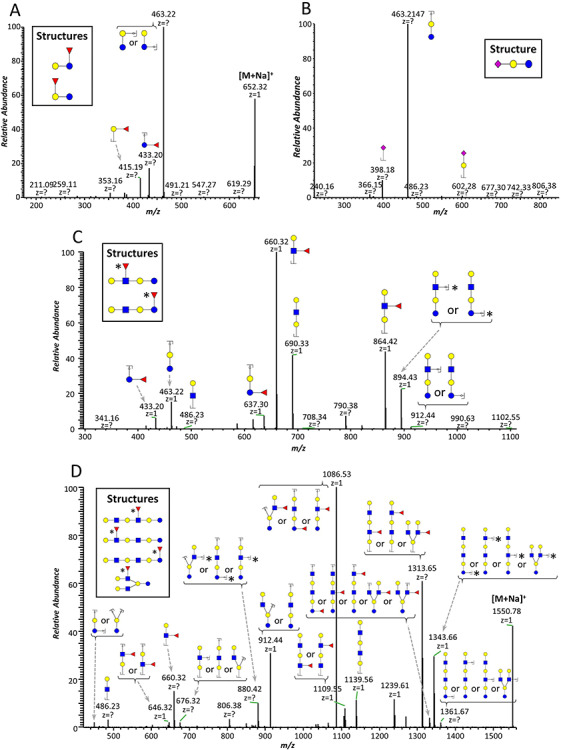
Structure assignment based on MS/MS analysis. CID MS^2^ spectra of (**A**) *m*/*z* 651.32, (**B**) *m*/*z* 838.40, (**C**) *m*/*z* 1100.55 and (**D**) *m*/*z* 1549.77. Permethylated fragments aid in structure determination and evaluation of number of isomers for a given mass. Fragments marked with asterisk represent fucose linkage isomers. This figure is available in black and white in print and in colour at *Glycobiology* online.

This type of transfer-related misassignment would be a particularly relevant problem for HMOs, where Lewis structures may be related to a number of biological functions including the determination of secretor status. Remarkably, permethylation was found to prevent this type of fucose migration reaction during MS/MS experiments (Aldredge et al. 2013; Zhou et al. 2017; Mank et al. 2019). Considering that many HMO isomers are related with fucose position, fucose migration during MS/MS fragmentation can severely hamper structure determination. Thus, MS/MS analysis of permethylated HMOs can assist in structure assignment without further enzymatic digestion of the glycan (Figure 5).

### Structure assignment and isomer differentiation based on MS/MS fragmentation

**Fig. 6:**
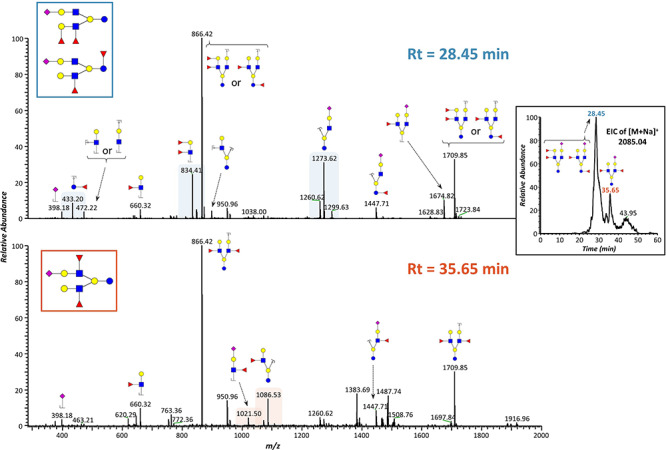
Isomer distinction based on chromatographic separation and MS/MS fragmentation. CID MS^2^ spectra of EIC peaks of [M + Na]^+^ 2085.04, eluting at 28.45 and 35.65 min. Characteristic fragments that allow for isomer(s) identification are highlighted. This figure is available in black and white in print and in colour at *Glycobiology* online.

Structural assignment of low molecular weight HMOs is fairly simple, considering the small number of fragment ions resulting from these glycans. For instance, in the case of *m*/*z* 651.32, we can detect two fucose position isomers of fucosyllactose, which can be distinguished by the fragments at *m*/*z* 415.19 corresponding to terminal fucosylation, and *m*/*z* 433.20 corresponding to core fucosylation (Figure 5A). In the case of sialylated HMOs, such as sialyllactose (*m*/*z* 838.40), it is more difficult to detect the presence of isomers because the glycosidic bonds linking sialic acid residues are especially labile, meaning that upon fragmentation, the neutral loss of Neu5Ac is the main fragment ion, as observed in Figure 5B. Hence, and although the EIC of this mass could suggest the presence of multiple peaks (Figure 3), the distinction between 2,3- and 2,6-linked sialic acids (which would be a source of isomerization) requires further MS^n^ fragmentation (Anthony et al. 2008). In many cases, HMOs can show isomerization of both position and linkage of fucose. This is illustrated in Figure 5C, where two fucose positional isomers can be observed for *m*/*z* 1100.55 (inset). These isomers can be distinguished by the fragments at *m*/*z* 433.20 and 637.30, which indicate core fucosylation, as well as fragments at *m*/*z* 660.32 and 864.42, which indicate the presence of a Lewis epitope. Aside from these positional isomers, Figure 5C also shows the presence of linkage isomers of fucose, as demonstrated by fragments at *m*/*z* 894.43 and 912.44, which correspond to the loss of a 4-linked fucose and a 3-linked fucose residue, respectively (Terada et al. 2005). With the increase in molecular weight, there is a proportional increase in glycoform structure complexity, which is distinctly demonstrated in Figure 5D. The MS/MS spectrum of *m*/*z* 1549.77 reveals both position and linkage isomers of fucose, as well as linear and branched structures, as suggested by the presence of a fragment at *m*/*z* 449.20. Thus, we can say that there are four possible core structures (inset) for this mass, and that each of these can potentially contain further fucose linkage isomers (*m*/*z* 1343.66). These results reflect the importance of proper chromatographic separation for isomer distinction. As discussed previously, using a common reversed-phase C18 column, co-elution of isomers is unavoidable (Dong et al. 2016; Mank et al. 2019), although some separation can be accomplished, as shown in Figure 6. Physical separation of glycoforms in the column facilitates analysis of MS/MS results, as peaks eluting at different times will have different MS^2^ spectra. Here we show the example of the HMO Hex_4_HexNAc_2_Fuc_2_NeuAc_1_, with mass 2085.04. Three isomers were found for this mass, two of them co-eluting at 28.5 min and the third eluting separately at 35.7 min. These isomers are distinguishable by their characteristic MS^2^ fragments (highlighted).

Although some studies have described isomer-specific CID fragments for the distinction of low molecular weight HMOs by MS^2^ analysis (Mank et al. 2019), MS^2^ fragmentation alone cannot provide enough unambiguous information to completely determine entire glycan structures, especially for HMOs of high molecular weight. Thus, we provide here examples of structural information that can be obtained from MS^2^ experiments, but full determination would require further MS^n^ experiments. For example, milk group determination would require up to MS^4^ experiments to distinguish fragments coming from different Lewis epitopes. However, in studies with high numbers of samples, full structure characterization is impractical, and often the goal of these studies is to compare milk profiles from different individuals, as samples from different donors can have very different HMO patterns (Ruhaak and Lebrilla 2012). In such cases, the approach presented here can be used as a profiling tool, providing information on the diversity and relative abundance of HMOs between subjects.

## Conclusions

HMO characterization is an analytical challenge as a result of the heterogeneity of molecular species that are typically present in a sample. We present here a method for milk oligosaccharide analysis, based on high-resolution chromatography and mass spectrometry. This method, which does not aim to be quantitative, takes advantage of instrumentation that is increasingly more accessible and allows for sensitive, high-resolution analysis of carbohydrate structures. Using reversed-phase separation of permethylated HMOs and CID MS/MS fragmentation, we identified over 100 HMOs masses, many of them displaying structural isomers. We also detected new HMOs of higher molecular weight than previously reported (permethylated mass 5317.67). We recognize that permethylation is not a perfect method. Permethylation can result in partially methylated products, prohibits the use of enzymes for structural analysis and diminishes the ability to perform extensive isomer separation. However, we believe the benefits of permethylation outweigh the shortcomings, which is why this is an established procedure in carbohydrate chemistry. We now hope to improve this method by introducing an internal standard to allow for full quantification of glycoforms and also plan to improve chromatographic separation of isomeric species. Furthermore, emerging software tools such as SimGlycan (Apte and Meitei 2010) and, more recently, GRITS toolbox (Weatherly et al. 2019), designed for archiving, processing and interpreting analytical data, can assist in structural characterization of HMOs, facilitating the development of new high-throughput methods. This, in addition to sample reduction to prevent peak splitting, would be another valuable improvement of the current method. In summary, we have developed a method for HMO analysis that has the advantage of providing structural data. This method, which can be further optimized into high-throughput, has a potential to be applied in screening of breast milk from mothers from different countries and accessing heterogeneity on HMO structural motifs from different regions and socioeconomical backgrounds. This method can also be used to screen milk oligosaccharides from other mammals and commercial products.

## Materials and methods

### Chemicals and reagents

Reagent water used throughout this study was obtained from a Barnstead™ Nanopure™ water purification system (Thermo-Fisher, Waltham, MA, USA). Solvents, LC-MS grade, were obtained from Thermo-Fisher Scientific (Waltham, MA, USA) and Sigma Aldrich (St. Louis, MO, USA) and were of the highest available quality.

### Milk samples

Human milk samples were from the PROVIDE birth cohort study carried out in Dhaka, Bangladesh by the International Centre for Diarrhoeal Disease Research, Bangladesh (icddr,b) (Kirkpatrick et al. 2015). Breast milk samples collected within 6 weeks post-partum from 130 mothers were used in this study. From this group of 130 samples, 6 were randomly selected and used for the current analysis.

### Sample preparation

Initial processing of samples was carried out at icddr,b. Breast milk samples were centrifuged in 15 mL Falcon tubes at 4,000 rpm for 10 min. After centrifugation, breast milk appears as three layers. The middle layer (defatted) was taken into a 1.5 mL micro centrifuge tube, then preservative (10% sodium azide and 0.1 M PMSF) was added at a concentration of 20 μL/mL, mixed and stored at −70°C. Samples were shipped in dry ice to the University of Georgia for analysis at the Complex Carbohydrate Research Center. Fifty microliters of defatted milk were diluted in half with water and subjected to ethanol precipitation (two volumes of ethanol, −80°C, 1 h) for protein removal. After 30 min of centrifugation at 14,000 rpm, 4°C, the upper liquid fraction was collected and lyophilized in preparation for permethylation. To the lyophilized HMOs (200 μg), dissolved in 200 μL of anhydrous dimethyl sulfoxide (DMSO), 300 μL of sodium hydroxide (NaOH) base in anhydrous DMSO (prepared separately by mixing NaOH and DMSO) was added and vortexed. A volume of 100 μL of CH_3_I was added to the sample, and the reaction mixture was vortexed vigorously for 10 min. The permethylation reaction was quenched by the addition of 2 mL of ddH_2_O, excess CH_3_I was bubbled off by a stream of nitrogen gas and glycans were purified by liquid-liquid extraction with dichloromethane. After solvent removal by a stream of nitrogen gas, the permethylated HMOs were dissolved in a final volume of 50 μL of methanol and filtered (0.2 μm).

### MALDI-TOF-MS analysis

A 1 μL aliquot of the permethylated HMOs was mixed with 1 μL of 2,5-dihydroxy benzoic acid (DHB) matrix (20 mg/mL in 1:1 methanol/ddH_2_O) and spotted on a MALDI plate. The samples were analyzed on a MALDI-TOF-MS instrument (AB SCIEX TOF/TOF 5800, Applied Biosystem MDS Analytical Technologies) in reflector positive ion mode. Structural annotation of glycan MALDI-TOF-MS data was performed manually and the masses were searched against the search engine Glycomod (https://web.expasy.org/glycomod/).

### nLC-NSI-MS/MS analysis

nLC-NSI-MS/MS analysis was performed using a Dionex UltiMate 3000 LC system coupled with Orbitrap Fusion Tribrid Mass Spectrometer (ThermoFisher) equipped with a nanospray ion source. Samples (2 μL) were injected onto an Acclaim PepMap™ 100 C18 column (75 μm × 15 cm, nanoViper) (ThermoFisher). Separation was performed using 2% acetonitrile, 0.1% formic acid, 1 mM sodium acetate as solvent A and 80% acetonitrile, 0.1% formic acid as solvent B. Oligosaccharides were eluted for 10 min with 20% B. In 1 min, solvent B was increased to 38%, and a 32 min linear gradient of solvent B was used to increase the percentage to 60%. Finally, a clean-up step was added at the end of the run and the conditions were returned to the original settings for 9 min. A constant flow rate of 0.3 μL/min was used. The mass spectrometer was operated in “top-down” data-dependent mode controlled by Thermo Xcalibur (version 3.0.63). This method selected the highest intensity ions within mass range for fragmentation based on the full mass spectra, collected as much data as possible within a 3 s window and excluded additional spectra after 2 within a 120 s window. Using a spray voltage of 1900V, a full FTMS spectrum was collected with the Orbitrap detector at a resolution of 120 000 at *m*/*z* from 400 to 2000. The ion transfer tube temperature was set to 275°C and MS analysis was performed in the positive ion mode. Product ion trigger analysis was performed using CID (collision energy (%): 35) and HCD (collision energy (%): 28). Structural annotation of glycan MS/MS data was performed manually and MS/MS fragments were assigned based on predicted theoretical fragments provided by GlycoWorkBench (Ceroni et al. 2008).

## Funding

National Institutes of Health (NIH)-funded Research Resource for Biomedical Glycomics (P41GM10349010); NIH–funded Orbitrap Fusion Tribrid Mass Spectrometer (1S10OD018530 to P.A.); Bill and Melinda Gates Foundation (OPP1173478): “Microbiome and Metabolomic Analysis of Selected PROVIDE Samples”.

## Conflict of interest statement

None declared.

## Supplementary Material

Supplementary_Data_final_cwaa028

## Abbreviations

CE, capillary electrophoresis; CID, collision-induced dissociation; EIC, extracted ion chromatogram; HILIC, hydrophilic interaction chromatography HPLC; HMOs, human milk oligosaccharides; HPAEC-PAD, high pH anion-exchange chromatography with pulsed amperometric detection; LC-MS/MS, liquid chromatography coupled with tandem mass spectrometry; MALDI-FTICR-MS, matrix-assisted laser-induced Fourier-transform ion cyclotron resonance mass spectrometry; MALDI-TOF-MS, matrix-assisted laser-induced time-of-flight mass spectrometry; MEKC, micellar electrokinetic chromatography; nLC-NSI-MS/MS, nanoliquid chromatography nanospray tandem mass spectrometry; nano-LC chip-TOF MS, nanoliquid chromatography chip time-of-flight mass spectrometry; PGC, porous graphitized carbon; RP-HPLC, reversed-phase high-performance liquid chromatography; TIC, total ion current
