# Supplementary material for: New strategies for profiling and characterization of human milk oligosaccharides
Source: Glycobiology. 2020 Apr 2;30(10):774–86. doi: 10.1093/glycob/cwaa028 (PMC7526734; doi:10.1093/glycob/cwaa028)
Supplement: Supplementary_Data_final_cwaa028 [file supplementary_data_final_cwaa028.docx]

**Supplementary Data**

**New strategies for profiling and characterization of human milk oligosaccharides**

Sara Porfirio^1^, Stephanie Archer-Hartmann^1^, G. Brett Moreau^2^, Girija Ramakrishnan^2^, Rashidul Haque^3^, Beth D. Kirkpatrick^4^, William A. Petri, Jr.^2^, Parastoo Azadi^1^

^1^ Complex Carbohydrate Research Center, The University of Georgia, Athens, GA 30602, USA

^2^ Dept. of Medicine/Infectious Diseases, University of Virginia, Charlottesville, VA 22903, USA

^3^ International Centre for Diarrhoeal Disease Research, Bangladesh (icddr,b), Dhaka 1212, Bangladesh

^4^ Dept. of Medicine, University of Vermont, Burlington, VT 05401, USA

**Table SI. HMOs detected in each human sample analyzed. Mass errors (ppm) for each HMO are indicated. Relative peak areas were calculated by dividing the peak area of the most abundant ion of each individual oligosaccharide over the total peak area of all HMOs detected. Most abundant charge states are shown in Table I.**

| **Composition** | **Theoretical mass** | **Sample 1** | | **Sample 2** | | **Sample 3** | | **Sample 4** | | **Sample 5** | | **Sample 6** | |
| --- | --- | --- | --- | --- | --- | --- | --- | --- | --- | --- | --- | --- | --- |
|  |  | **Δmass (ppm)** | **Peak area %** | **Δmass (ppm)** | **Peak area %** | **Δmass (ppm)** | **Peak area %** | **Δmass (ppm)** | **Peak area %** | **Δmass (ppm)** | **Peak area %** | **Δmass (ppm)** | **Peak area %** |
| Hex_2_ Fuc_1_ | 651.3200 | 1.277 | 14.60 | 0.970 | 13.75 | 0.817 | 12.17 | 1.124 | 12.58 | 0.970 | 8.41 | 0.663 | 13.16 |
| Hex_2_ HexNAc_1_ | 722.3572 | 1.706 | 0.09 | 1.013 | 0.08 | 0.598 | 0.15 | 1.290 | 0.05 | 0.875 | 0.17 | 0.736 | 0.03 |
| Hex_2_ Fuc_2_ | 825.4092 | 1.735 | 0.84 | 1.735 | 0.98 | 1.371 | 0.40 | - | - | 1.008 | 0.68 | 0.887 | 1.65 |
| Hex_2_ NeuAc_1_ | 838.4045 | 2.066 | 16.37 | 1.827 | 17.41 | 1.589 | 16.52 | 1.947 | 12.69 | 1.947 | 6.33 | 0.157 | 23.10 |
| Hex_2_ HexNAc_1_ Fuc_1_ | 896.4464 | 1.263 | 0.03 | 1.932 | 0.08 | 0.928 | 0.04 | 1.486 | 0.05 | 1.486 | 0.05 | 0.928 | 0.05 |
| Hex_3_ HexNAc_1_ | 926.4570 | 0.358 | 8.15 | 0.358 | 9.08 | 0.142 | 8.11 | 0.142 | 0.45 | 0.142 | 6.60 | 0.358 | 3.01 |
| Hex_3_ HexNAc_1_ Fuc_1_ | 1100.5462 | 0.665 | 11.02 | 0.483 | 12.32 | 0.302 | 8.53 | 0.483 | 14.71 | 0.665 | 9.04 | 0.483 | 8.10 |
| Hex_4_ HexNAc_1_ | 1130.5568 | 0.471 | 0.50 | 0.647 | 0.47 | 0.117 | 0.42 | -0.059 | 1.11 | 0.294 | 0.42 | 0.647 | 0.27 |
| Hex_3_ HexNAc_1_ Fuc_2_ | 1274.6354 | 1.359 | 5.56 | 1.437 | 4.70 | 1.045 | 2.50 | 1.202 | 6.07 | 1.359 | 5.62 | 1.359 | 5.64 |
| Hex_3_ HexNAc_1_ NeuAc_1_ | 1287.6307 | 1.500 | 2.16 | 1.345 | 3.33 | 1.345 | 3.08 | 1.423 | 2.47 | 1.500 | 1.52 | 1.190 | 2.39 |
| Hex_4_ HexNAc_1_ Fuc_1_ | 1304.6460 | 1.174 | 0.39 | 1.021 | 0.52 | 0.868 | 0.28 | 0.868 | 1.79 | 1.251 | 1.25 | 0.714 | 0.34 |
| Hex_3_ HexNAc_2_ Fuc_1_ | 1345.6726 | 1.584 | 0.04 | 2.253 | 0.08 | 1.436 | 0.07 | 1.213 | 0.10 | 0.916 | 0.07 | 1.361 | 0.07 |
| Hex_4_ HexNAc_2_ | 1375.6832 | 1.114 | 0.90 | 1.114 | 0.54 | 0.677 | 0.67 | 0.823 | 0.44 | 0.968 | 0.61 | 0.823 | 0.22 |
| Hex_3_ HexNAc_1_ Fuc_1_ NeuAc_1_ | 1461.7199 | 1.459 | 0.08 | 1.048 | 0.45 | 1.185 | 0.35 | 0.911 | 1.20 | 1.459 | 0.68 | 0.980 | 0.18 |
| Hex_4_ HexNAc_1_ Fuc_2_ | 1478.7352 | 1.171 | 0.05 | 0.901 | 0.16 | 1.036 | 0.03 | - | - | 1.374 | 1.98 | 1.307 | 0.11 |
| Hex_4_ HexNAc_1_ NeuAc_1_ | 1491.7305 | - | - | 1.630 | 0.18 | 1.027 | 0.02 | 1.429 | 0.15 | - | - | 1.429 | 0.03 |
| Hex_3_ HexNAc_2_ Fuc_2_ | 1519.7618 | - | - | - | - | - | - | 1.008 | 0.04 | - | - | - | - |
| Hex_4_ HexNAc_2_ Fuc_1_ | 1549.7724 | 1.311 | 3.07 | 1.053 | 3.47 | 0.924 | 2.60 | 0.924 | 3.08 | 1.053 | 3.30 | 1.311 | 1.70 |
| Hex_5_ HexNAc_2_ | 1579.7830 | 1.033 | 0.12 | - | - | 1.223 | 0.10 | 0.653 | 0.12 | 1.033 | 0.10 | - | - |
| Hex_3_ HexNAc_1_ NeuAc_2_ | 1648.8045 | 1.111 | 4.26 | 1.596 | 1.71 | 0.869 | 1.92 | 0.929 | 3.95 | 1.657 | 4.46 | 1.475 | 2.44 |
| Hex_3_ HexNAc_2_ Fuc_1_ NeuAc_1_ | 1706.8464 | - | - | - | - | - | - | - | - | - | - | 1.366 | 0.06 |
| Hex_4_ HexNAc_2_ Fuc_2_ | 1723.8617 | 1.469 | 4.32 | 1.295 | 5.37 | 1.237 | 3.04 | 1.121 | 8.20 | 1.411 | 4.36 | 1.353 | 3.46 |
| Hex_4_ HexNAc_2_ NeuAc_1_ | 1736.8569 | 0.882 | 1.65 | 1.055 | 0.93 | 0.767 | 0.81 | 0.824 | 0.66 | 0.940 | 0.72 | 0.997 | 0.82 |
| Hex_5_ HexNAc_2_ Fuc_1_ | 1753.8722 | 1.045 | 0.22 | 2.242 | 0.17 | 1.159 | 0.17 | 1.102 | 0.62 | 0.988 | 0.24 | 1.444 | 0.14 |
| Hex_3_ HexNAc_1_ Fuc_1_ NeuAc_2_ | 1822.8937 | 1.828 | 0.11 | 1.828 | 0.08 | 1.828 | 0.08 | 1.170 | 0.83 | 1.334 | 0.26 | 1.224 | 0.08 |
| Hex_5_ HexNAc_3_ | 1824.9094 | 0.401 | 0.23 | - | - | - | - | - | - | -0.968 | 0.28 | - | - |
| Hex_4_ HexNAc_2_ Fuc_3_ | 1897.9509 | 1.334 | 2.95 | -1.141 | 2.63 | 0.913 | 1.12 | 0.228 | 0.42 | 1.229 | 2.38 | 1.123 | 3.34 |
| Hex_4_ HexNAc_2_ Fuc_1_ NeuAc_1_ | 1910.9462 | 1.011 | 4.59 | 1.011 | 7.68 | 0.802 | 3.71 | 0.854 | 7.01 | 0.854 | 3.70 | 0.697 | 7.34 |
| Hex_5_ HexNAc_2_ Fuc_2_ | 1927.9615 | - | - | - | - | 1.106 | 0.05 | 0.535 | 0.33 | 0.535 | 0.56 | 2.143 | 0.19 |
| Hex_5_ HexNAc_2_ NeuAc_1_ | 1940.9567 | 1.408 | 0.19 | 2.232 | 0.23 | 1.408 | 0.16 | 1.098 | 0.37 | 1.820 | 0.13 | 1.253 | 0.30 |
| Hex_5_ HexNAc_3_ Fuc_1_ | 1998.9986 | 1.267 | 1.04 | 1.167 | 0.93 | 0.966 | 0.75 | 0.866 | 0.97 | 1.167 | 1.99 | 1.067 | 0.35 |
| Hex_4_ HexNAc_2_ Fuc_2_ NeuAc_1_ | 2085.0354 | 1.118 | 1.29 | 0.447 | 1.21 | 0.735 | 0.90 | 1.023 | 0.66 | 0.214 | 1.05 | 0.735 | 1.42 |
| Hex_4_ HexNAc_2_ NeuAc_2_ | 2098.0307 | 1.588 | 1.05 | 1.683 | 0.78 | 1.493 | 9.21 | 1.397 | 0.66 | 1.683 | 0.88 | 1.016 | 1.10 |
| Hex_5_ HexNAc_2_ Fuc_3_ | 2102.0507 | - | - | - | - | - | - | - | - | 1.205 | 1.09 | - | - |
| Hex_5_ HexNAc_2_ Fuc_1_ NeuAc_1_ | 2115.0460 | - | - | - | - | - | - | 0.913 | 0.12 | 0.630 | 0.07 | - | - |
| Hex_5_ HexNAc_3_ Fuc_2_ | 2173.0879 | 1.533 | 2.16 | 0.981 | 2.04 | 1.395 | 1.84 | 0.705 | 3.27 | 1.395 | 3.70 | 1.073 | 1.21 |
| Hex_6_ HexNAc_3_ Fuc_1_ | 2203.0984 | 1.059 | 0.12 | - | - | 0.786 | 0.10 | 1.059 | 0.30 | 0.968 | 0.21 | 1.422 | 0.05 |
| Hex_4_ HexNAc_2_ Fuc_1_ NeuAc_2_ | 2272.1199 | 1.114 | 1.72 | 1.290 | 1.42 | 1.246 | 2.28 | 1.114 | 2.23 | 1.246 | 2.78 | 1.026 | 3.13 |
| Hex_5_ HexNAc_3_ Fuc_3_ | 2347.1771 | 0.738 | 1.71 | -0.369 | 1.64 | 0.482 | 1.38 | 0.567 | 2.63 | 0.653 | 2.92 | 0.312 | 1.51 |
| Hex_5_ HexNAc_3_ Fuc_1_ NeuAc_1_ | 2360.1724 | -0.028 | 0.46 | 0.649 | 0.53 | 0.056 | 0.75 | 0.649 | 0.52 | 0.564 | 0.73 | 0.480 | 0.43 |
| Hex_6_ HexNAc_3_ Fuc_2_ | 2377.1877 | 0.644 | 0.11 | - | - | 0.644 | 0.08 | 0.813 | 0.31 | 0.897 | 0.48 | 1.065 | 0.07 |
| Hex_6_ HexNAc_4_ Fuc_1_ | 2448.2249 | 0.953 | 0.98 | 1.034 | 0.82 | 0.953 | 1.79 | 0.871 | 1.11 | 1.034 | 2.20 | 0.544 | 0.64 |
| Hex_4_ HexNAc_2_ NeuAc_3_ | 2459.2044 | 0.135 | 0.23 | 1.030 | 0.15 | 0.216 | 0.56 | 0.867 | 0.23 | 1.355 | 0.32 | 0.786 | 0.72 |
| Hex_5_ HexNAc_3_ Fuc_4_ | 2521.2664 | 0.766 | 0.75 | - | - | 0.290 | 0.54 | - | - | 0.885 | 1.37 | 0.687 | 0.86 |
| Hex_5_ HexNAc_3_ Fuc2 NeuAc_1_ | 2534.2616 | 1.512 | 0.50 | 1.394 | 0.64 | 0.920 | 1.21 | 1.275 | 1.07 | 0.447 | 1.07 | 1.394 | 0.79 |
| Hex_5_ HexNAc_3_ NeuAc_2_ | 2547.2569 | 0.287 | 0.06 | 1.465 | 0.01 | 0.601 | 0.07 | 0.130 | 0.04 | 0.994 | 0.17 | 0.758 | 0.03 |
| Hex_6_ HexNAc_4_ Fuc_2_ | 2622.3141 | 1.118 | 0.98 | 0.813 | 1.01 | 1.271 | 2.10 | 1.118 | 1.07 | 1.347 | 2.22 | 1.194 | 1.00 |
| Hex_6_ HexNAc_4_ NeuAc_1_ | 2635.3094 | 0.430 | 0.09 | 0.961 | 0.08 | 0.809 | 0.28 | 0.733 | 0.08 | 0.202 | 0.18 | 1.037 | 0.08 |
| Hex_5_ HexNAc_3_ Fuc_5_ | 2695.3556 | 0.568 | 0.13 | - | - | -0.247 | 0.07 | - | - | -0.024 | 0.31 | 0.531 | 0.25 |
| Hex_5_ HexNAc_3_ Fuc_3_ NeuAc_1_ | 2708.3509 | 0.787 | 0.19 | -1.427 | 0.35 | 0.344 | 0.47 | -0.098 | 0.18 | 0.344 | 0.65 | 0.566 | 0.54 |
| Hex_5_ HexNAc_3_ Fuc_1_ NeuAc_2_ | 2721.3461 | 0.342 | 0.21 | 0.563 | 0.15 | -0.024 | 0.44 | 0.636 | 0.26 | 0.636 | 0.62 | 0.636 | 0.27 |
| Hex_6_ HexNAc_4_ Fuc_3_ | 2796.4033 | 1.084 | 0.58 | -1.919 | 0.76 | 1.192 | 1.29 | 0.762 | 0.89 | 1.120 | 1.55 | 0.870 | 0.97 |
| Hex_6_ HexNAc_4_ Fuc_1_ NeuAc_1_ | 2809.3986 | 0.688 | 0.17 | 1.186 | 0.20 | 0.759 | 0.81 | 0.901 | 0.22 | 0.545 | 0.42 | 1.328 | 0.31 |
| Hex_5_ HexNAc_3_ Fuc_2_ NeuAc_2_ | 2895.4354 | 0.529 | 0.22 | 0.529 | 0.09 | 0.460 | 0.41 | 1.013 | 0.38 | 0.874 | 0.73 | 0.529 | 0.28 |
| Hex_6_ HexNAc_4_ Fuc_4_ | 2970.4926 | 0.246 | 0.25 | - | - | 0.145 | 0.47 | 0.347 | 0.23 | -0.662 | 0.65 | 0.516 | 0.51 |
| Hex_6_ HexNAc_4_ Fuc_2_ NeuAc_1_ | 2983.4878 | -0.022 | 0.15 | 1.184 | 0.18 | 1.117 | 0.73 | 0.983 | 0.27 | -0.022 | 0.44 | 1.284 | 0.42 |
| Hex_6_ HexNAc_4_ NeuAc_2_ | 2996.4831 | 0.712 | 0.02 | - | - | 0.645 | 0.14 | 0.912 | 0.01 | 1.412 | 0.09 | 1.212 | 0.01 |
| Hex_5_ HexNAc_3_ Fuc_3_ NeuAc_2_ | 3069.5246 | 0.857 | 0.09 | 1.281 | 0.03 | 0.629 | 0.15 | 0.043 | 0.02 | 0.760 | 0.37 | 0.955 | 0.21 |
| Hex_7_ HexNAc_5_ Fuc_2_ | 3071.5403 | 0.824 | 0.24 | - | - | - | - | 0.271 | 0.19 | - | - | - | - |
| Hex_5_ HexNAc_3_ Fuc_1_ NeuAc_3_ | 3082.5199 | 0.789 | 0.04 | - | - | 1.276 | 0.05 | - | - | - | - | 0.984 | 0.08 |
| Hex_6_ HexNAc_3_ Fuc_2_ NeuAc_2_ | 3099.5352 | 0.301 | 0.01 | - | - | - | - | - | - | 0.688 | 0.08 | 0.881 | 0.02 |
| Hex_6_ HexNAc_4_ Fuc_5_ | 3144.5818 | 0.360 | 0.13 | - | - | - | - | - | - | 0.551 | 0.75 | 0.455 | 0.28 |
| Hex_6_ HexNAc_4_ Fuc_3_ NeuAc_1_ | 3157.5771 | 0.359 | 0.21 | - | - | 1.055 | 0.31 | 0.549 | 0.21 | 0.390 | 0.29 | 0.137 | 0.50 |
| Hex_6_ HexNAc_4_ Fuc_1_ NeuAc_2_ | 3170.5724 | 0.799 | 0.26 | 0.672 | 0.03 | 0.956 | 0.36 | 1.429 | 0.22 | 0.988 | 0.35 | 0.956 | 0.28 |
| Hex_7_ HexNAc_5_ Fuc_3_ | 3245.6296 | 0.564 | 0.33 | 0.657 | 0.22 | 0.842 | 0.39 | 0.842 | 0.42 | 0.842 | 0.65 | 0.657 | 0.26 |
| Hex_5_ HexNAc_3_ NeuAc_4_ | 3269.6044 | 0.958 | 0.04 | - | - | 1.233 | 0.01 | - | - | 1.050 | 0.05 | - | - |
| Hex_6_ HexNAc_3_ Fuc_3_ NeuAc_2_ | 3273.6244 | - | - | - | - | - | - | - | - | 0.407 | 0.14 | - | - |
| Hex_8_ HexNAc_5_ Fuc_2_ | 3275.6401 | - | - | - | - | 0.437 | 0.03 | 0.285 | 0.05 | - | - | - | - |
| Hex_6_ HexNAc_4_ Fuc_4_ NeuAc_1_ | 3331.6663 | -2.331 | 0.07 | - | - | - | - | - | - | -0.500 | 0.12 | - | - |
| Hex_6_ HexNAc_4_ Fuc_2_ NeuAc_2_ | 3344.6616 | 0.219 | 0.27 | 0.398 | 0.10 | 0.757 | 0.48 | 0.757 | 0.35 | 0.129 | 0.53 | 0.757 | 0.42 |
| Hex_7_ HexNAc_5_ Fuc_4_ | 3419.7188 | 0.857 | 0.16 | - | - | 0.828 | 0.25 | 0.828 | 0.30 | 0.740 | 0.44 | 0.565 | 0.21 |
| Hex_7_ HexNAc_5_ Fuc_2_ NeuAc_1_ | 3432.7141 | 0.330 | 0.10 | 1.116 | 0.07 | 1.378 | 0.31 | 0.767 | 0.13 | 1.204 | 0.27 | 1.116 | 0.11 |
| Hex_5_ HexNAc_3_ Fuc_1_ NeuAc_4_ | 3443.6936 | 0.416 | 0.03 | - | - | 0.764 | 0.05 | 0.329 | 0.04 | 0.590 | 0.12 | 0.416 | 0.07 |
| Hex_8_ HexNAc_5_ Fuc_3_ | 3449.7294 | - | - | - | - | - | - | -0.106 | 0.04 | 0.328 | 0.19 | - | - |
| Hex_6_ HexNAc_4_ Fuc_3_ NeuAc_2_ | 3518.7508 | 0.151 | 0.10 | -0.786 | 0.04 | 0.322 | 0.20 | -0.359 | 0.08 | 0.236 | 0.29 | 0.549 | 0.29 |
| Hex_6_ HexNAc_4_ Fuc_1_ NeuAc_3_ | 3531.7461 | 2.642 | 0.03 | - | - | 0.774 | 0.15 | 0.887 | 0.06 | 2.812 | 0.07 | -0.358 | 0.12 |
| Hex_7_ HexNAc_5_ Fuc_5_ | 3593.8080 | -0.101 | 0.05 | - | - | 0.232 | 0.08 | -0.268 | 0.05 | 0.232 | 0.21 | 0.482 | 1.39 |
| Hex_9_ HexNAc_4_ Fuc_2_ NeuAc_1_ | 3595.7873 | - | - | -0.268 | 0.09 | - | - | - | - | - | - | - | - |
| Hex_7_ HexNAc_5_ Fuc_3_ NeuAc_1_ | 3606.8033 | 0.924 | 0.05 | 0.009 | 0.05 | 1.400 | 0.26 | 1.201 | 0.13 | 1.922 | 0.20 | 1.700 | 0.13 |
| Hex_5_ HexNAc_3_ Fuc_2_ NeuAc_4_ | 3617.7829 | - | - | - | - | - | - | -0.156 | 0.01 | - | - | - | - |
| Hex_7_ HexNAc_5_ Fuc_1_ NeuAc_2_ | 3619.7986 | 0.589 | 0.03 | - | - | 0.755 | 0.11 | - | - | 0.423 | 0.14 | - | - |
| Hex_6_ HexNAc_4_ Fuc_4_ NeuAc_2_ | 3692.8401 | 0.577 | 0.01 | - | - | - | - | - | - | - | - | 0.902 | 0.04 |
| Hex_8_ HexNAc_6_ Fuc_3_ | 3694.8558 | - | - | - | - | - | - | 0.144 | 0.15 | 0.415 | 0.46 | - | - |
| Hex_6_ HexNAc_4_ Fuc_2_ NeuAc_3_ | 3705.8354 | - | - | - | - | 0.980 | 0.05 | -0.071 | 0.04 | 1.547 | 0.04 | 0.575 | 0.04 |
| Hex_6_ HexNAc_4_ NeuAc_4_ | 3718.8306 | 2.133 | 0.01 | - | - | 1.084 | 0.02 | - | - | 1.730 | 0.04 | 2.294 | 0.01 |
| Hex_7_ HexNAc_5_ Fuc_2_ NeuAc_2_ | 3793.8878 | 0.246 | 0.05 | - | - | - | - | -0.491 | 0.08 | 0.298 | 0.25 | - | - |
| Hex_8_ HexNAc_6_ Fuc_4_ | 3868.9450 | 0.396 | 0.05 | - | - | 1.120 | 0.24 | 0.706 | 0.07 | 0.758 | 0.26 | 0.086 | 0.10 |
| Hex_8_ HexNAc_6_ Fuc_2_ NeuAc_1_ | 3881.9403 | 0.060 | 0.02 | - | - | 1.296 | 0.19 | 0.910 | 0.04 | 0.678 | 0.12 | 0.137 | 0.04 |
| Hex_6_ HexNAc_4_ Fuc_1_ NeuAc_4_ | 3892.9199 | - | - | - | - | 1.909 | 0.04 | 1.138 | 0.01 | 1.061 | 0.03 | 0.368 | 0.02 |
| Hex_7_ HexNAc_5_ Fuc_3_ NeuAc_2_ | 3967.9771 | 0.815 | 0.02 | - | - | 1.268 | 0.13 | 1.419 | 0.05 | 0.789 | 0.18 | 0.966 | 0.06 |
| Hex_8_ HexNAc_6_ Fuc_5_ | 4043.0343 | - | - | - | - | - | - | -0.758 | 0.02 | 0.132 | 0.11 | -0.758 | 0.05 |
| Hex_8_ HexNAc_6_ Fuc_3_ NeuAc_1_ | 4056.0295 | - | - | - | - | -0.534 | 0.12 | 1.832 | 0.02 | 0.353 | 0.06 | -0.829 | 0.03 |
| Hex_7_ HexNAc_5_ Fuc_4_ NeuAc_2_ | 4142.0663 | - | - | - | - | 0.949 | 0.02 | - | - | -0.353 | 0.07 | 0.442 | 0.03 |
| Hex_8_ HexNAc_6_ Fuc_6_ | 4217.1235 | - | - | - | - | - | - | - | - | 0.150 | 0.03 | 0.577 | 0.01 |
| Hex_8_ HexNAc_6_ Fuc_4_ NeuAc_1_ | 4230.1188 | - | - | - | - | 0.717 | 0.03 | - | - | -0.110 | 0.03 | -0.110 | 0.02 |
| Hex_8_ HexNAc_6_ Fuc_2_ NeuAc_2_ | 4243.1140 | - | - | - | - | 1.374 | 0.07 | 2.765 | 0.01 | 1.162 | 0.05 | 0.455 | 0.01 |
| Hex_8_ HexNAc_6_ Fuc_3_ NeuAc_2_ | 4417.2033 | - | - | - | - | 2.022 | 0.03 | - | - | 1.252 | 0.04 | - | - |
| Hex_9_ HexNAc_7_ Fuc_2_ NeuAc_2_ | 4692.3403 | - | - | - | - | - | - | - | - | -0.397 | 0.02 | - | - |
| Hex_10_ HexNAc_8_ Fuc_4_ | 4767.3975 | - | - | - | - | - | - | - | - | -0.664 | 0.03 | - | - |
| Hex_11_ HexNAc_9_ Fuc_2_ | 4868.4452 | - | - | - | - | - | - | - | - | -0.752 | 0.06 | - | - |
| Hex_11_ HexNAc_9_ Fuc_3_ | 5042.5344 | - | - | - | - | 0.264 | 0.04 | - | - | -0.052 | 0.04 | - | - |
| Hex_12_ HexNAc_10_ Fuc_2_ | 5317.6714 | - | - | - | - | -0.914 | 0.06 | - | - | - | - | - | - |
